# Effects of Dynamic Stability Training with Water Inertia Load on Gait and Biomechanics in Older Women: A Randomized Clinical Trial

**DOI:** 10.3390/jfmk10020207

**Published:** 2025-06-03

**Authors:** Hyun Ju Kim, Il Bong Park

**Affiliations:** Department of Sports Health Convergence, Busan University of Foreign Studies, Busan 46234, Republic of Korea; fnjboss@bufs.ac.kr

**Keywords:** gait, aged, female, exercise therapy, ankle joint, biomechanical phenomena

## Abstract

**Background:** Gait decline in older adults is closely linked to reduced ankle propulsion and a compensatory reliance on proximal joints. This randomized trial investigated whether dynamic stability training using water inertia can improve gait mechanics and redistribute lower-limb joint moments in older women. **Hypotheses:** (1) The training would improve gait speed, stride length, and cadence. (2) The ankle plantar flexor moment and positive mechanical work would increase, while hip extension moment would decrease. **Method:** Twenty-four women aged 65 years and older were randomly assigned to either an experimental or control group. The experimental group wore a water-filled aquavest, and the control group wore a weighted vest. Both groups performed the same training program twice weekly for 12 weeks. Outcome measures included gait speed, stride length, cadence, ankle plantar flexion moment, hip extension moment, and positive mechanical work during terminal stance. A two-way mixed (between–within) analysis of variance (ANOVA) evaluated the group × time interaction effects. **Results:** Significant group × time interactions were found for gait speed (*p* < 0.001), stride length (*p* < 0.001), ankle moment (*p* = 0.017), and positive work (*p* < 0.001). Cadence increased in both groups over time (*p* < 0.05), with no interaction. The hip moment declined slightly in the experimental group. **Conclusions:** Water inertia load training enhanced propulsion and promoted an ankle-dominant gait, supporting its use to improve gait function and reduce proximal compensation in older women.

## 1. Introduction

Aging is accompanied by a progressive decline in neuromuscular function, which leads to changes in gait patterns and an increased risk of falls [1,2,3]. In particular, reduced ankle strength and diminished proprioceptive sensitivity contribute to a decreased propulsive force during a terminal stance, which not only slows walking speed but also increases energy expenditure [4,5,6]. To compensate for this, older adults tend to rely more on proximal joints, such as the hip, further reducing gait efficiency [7,8].

Among older adults, women are at greater risk. Postmenopausal estrogen decline accelerates the loss of muscle mass and strength, making older women more vulnerable to gait dysfunction and falls compared to men [9,10]. Furthermore, from the age of 60, the decline in walking speed accelerates, and gait efficiency significantly deteriorates [11,12].

According to previous studies, restoring ankle strategy—namely generating propulsive force from distal segments—is critical for maintaining an efficient gait in older adults [8,13]. However, traditional resistance or balance training programs, often performed in stable environments, lack the sensory–motor integration stimuli required for actual walking, limiting their ability to induce neuromuscular adaptations or functional joint-level changes [14,15].

Water inertia training has recently gained attention as a novel approach for inducing dynamic, task-specific perturbations. This method involves carrying water-filled devices that generate internal, shifting loads, stimulating proprioceptive feedback and reactive control mechanisms [16,17]. Repeated exposure to such perturbations may enhance coordination and ankle stability, potentially increasing propulsion during gait [18,19,20].

Despite its potential, little is known about the biomechanical effects of water-inertia-based training—particularly on joint moment redistribution and positive mechanical work during gait. Here, joint moment redistribution refers to the altered mechanical contributions between the ankle and hip joints in response to impaired distal propulsion. While this concept has been explored in compensatory gait strategies, it remains unclear as to whether targeted interventions can reverse such redistribution in elderly populations.

Improvements in ankle function play a key role in reducing hip-dominant compensatory strategies that are commonly observed in older adults with diminished distal propulsion. Mechanistic studies have shown that increasing ankle power output can reduce the workload on the hip extensors and help shift joint moment distribution back toward the distal joints during gait [21,22]. Although direct evidence on the effects of water-based perturbation training on ankle power or positive mechanical work remains limited, a recent study reported that such training significantly increased the ankle range of motion during stair ambulation [17]. These findings suggest that the ankle is biomechanically responsive to perturbation stimuli generated by water inertia and that repeated exposure may promote adaptive improvements in distal joint function.

Importantly, water-inertia-based training differs fundamentally from traditional unstable-surface training. The latter typically employs tools such as foam pads or wobble boards, which may effectively stimulate proprioceptive feedback and challenge balance control. However, these tools provide an environment that differs from actual ground-contact conditions, resulting in the inconsistent transmission of ground reaction forces (GRF) and limiting the ability to accurately replicate gait-like movements [23,24,25].

In contrast, the present study implemented exercises on firm ground while participants wore a water-filled aquavest, thereby maintaining GRF conditions similar to those of real walking. Simultaneously, the shifting water mass introduced unpredictable, multi-directional internal perturbations. This setup offers a novel and task-relevant approach that preserves gait-related biomechanics while delivering reactive neuromuscular challenges applicable to daily life.

Accordingly, this study investigated the effects of a 12-week dynamic stability training program using water inertia load on spatiotemporal gait characteristics and lower-limb biomechanical variables in elderly women, with the goal of delivering realistic and task-relevant perturbation stimuli that simulate actual walking conditions.

The hypotheses of this study are as follows:

(1) The training will improve spatiotemporal parameters such as gait speed, stride length, and cadence.
(2) The ankle plantar flexor moment and positive mechanical work will increase, while compensatory hip moment will decrease.

These outcomes are expected to reflect the recovery of a more efficient propulsion strategy during gait.

## 2. Materials and Methods

### 2.1. Participants

A total of 30 healthy elderly women aged 65 years and older were recruited for this study. Recruitment was conducted through a community center and a university located in region B. Prior to participation, all individuals received a thorough explanation of the study’s purpose and procedures, and signed written informed consent was obtained. This study was approved by the Institutional Review Board (IRB approval number: P01-202409-01-034) and was prospectively registered at ClinicalTrials.gov (Identifier: NCT06705946) on 25 November 2024.

Participants were randomly assigned to either the experimental group (n = 12) or the control group (n = 12). Simple randomization was conducted using a pre-generated list of random numbers, which were placed in sequentially numbered, opaque, sealed envelopes. These envelopes were opened consecutively following each participant’s enrollment. The random sequence was generated by the lead researcher, who also enrolled participants and assigned them to groups.

During the intervention period, six participants withdrew due to health-related reasons, including minor injuries sustained in daily life and concerns regarding pre-existing medical conditions. Consequently, the final analysis included data from 24 participants, with 12 individuals in each group.

Outcome assessments were conducted by the same researcher who delivered the intervention and was thus aware of group assignments. To minimize potential bias, all measurements followed standardized protocols and were applied consistently to all participants. Data analysis was performed only after the completion of all data collection. Participants were blinded to the group allocation and were not informed of the specific hypotheses being tested.

The required sample size was calculated using G*Power 3.1 software for a mixed-design repeated-measures ANOVA (2 groups × 3 time points), with a focus on interaction effects. An effect size of *f* = 0.32 was adopted based on a previous study implementing a similar inertial load of a water training program in older adults [18]. With an alpha level of 0.05 and statistical power (1 − β) = 0.80, the analysis indicated that a minimum of 18 participants would be required. Considering the dropout rates of 20–25% reported in prior intervention studies involving elderly populations, a total of 30 participants were initially recruited [26]. With 24 participants included in the final analysis, the study retained sufficient statistical power to detect the expected effects.

The inclusion criteria were as follows:

(1) Healthy women aged 65 years or older

The exclusion criteria were as follows:

(1) History of musculoskeletal disorders within the past 3 months
(2) Severe cardiopulmonary diseases (e.g., heart failure, myocardial infarction)
(3) Use of medications such as anxiolytics, antidepressants, or sedatives
(4) Diagnosis of chronic pulmonary disease
(5) History of surgery within the past 6 months.

**Figure 1:**
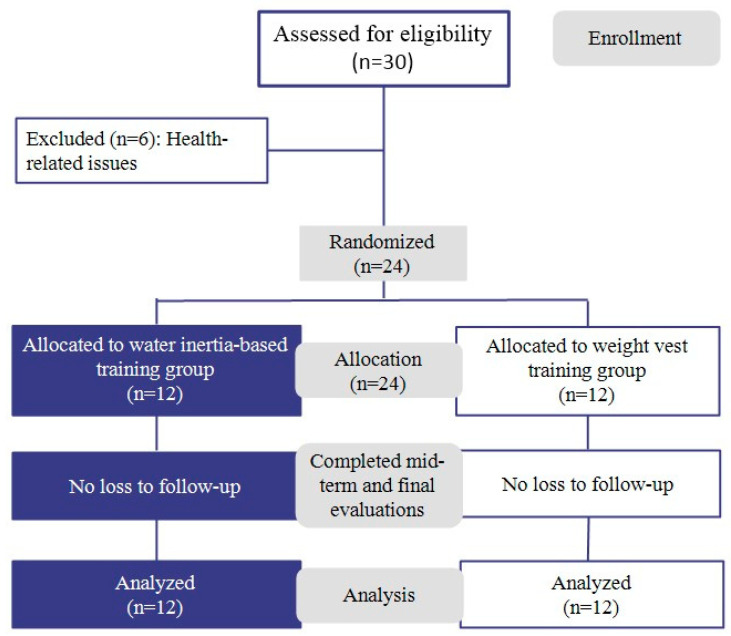
Flow diagram of the study participants.

Furthermore, homogeneity tests confirmed that there were no significant differences between the groups in terms of age (t = −0.676), height (t = 0.573), weight (t = 0.810), or BMI (t = −0.557), indicating that the baseline physical characteristics were statistically comparable. Additionally, all participants confirmed that they had not engaged in any structured physical activity programs within the six months preceding the study. Table 1 and Figure 1 present the participants’ general characteristics and the flow diagram of participant allocation throughout the study.

### 2.2. Gait Assessment and Data Acquisition

**Figure 2:**
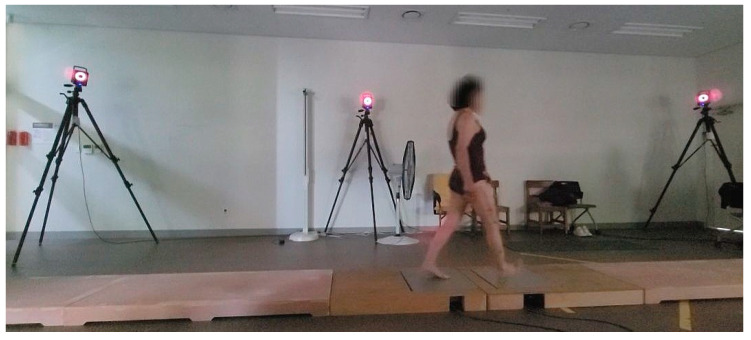
3D motion capture system and 6 m walkway.

Spatiotemporal gait parameters such as gait speed, stride length, and cadence are important indicators of functional mobility and fall risk in older adults [27,28]. Accordingly, to assess these parameters, a 6-meter walkway and six infrared-based three-dimensional motion capture cameras (Vicon camera MX-T20, Oxford Metrics, Oxford, UK) were utilized, as shown in Figure 2 [29].

Gait assessment and data acquisition were conducted by the first author, who had received two years of specialized training in gait analysis under the supervision of a faculty expert in biomechanics. The researcher had also participated in previous studies involving motion capture and gait assessment, further contributing to consistency and accuracy in the data collection.

Gait measurements were conducted in a quiet environment with minimal external distractions. Prior to data collection, participants performed familiarization trials to adapt to the testing protocol. Each participant walked barefoot at a self-selected, comfortable pace, and six trials were recorded. From these trials, three representative and artifact-free gait cycles were selected per participant based on accurate foot placement and signal quality. To enhance measurement reliability and detect subtle changes in gait velocity, all assessments were repeated under identical conditions [30]. All participants wore swimwear to minimize clothing interference with the markers, and measurements were performed barefoot [31].

All kinematic and kinetic data were collected and processed using Vicon Nexus v2.15 (Oxford Metrics, UK) with a sampling rate of 100 Hz. Marker trajectory data were automatically filtered using a Woltring filter (generalized cross-validatory spline smoothing) embedded in the Plug-in Gait pipeline. GRF data were filtered using a fourth-order zero-lag Butterworth low-pass filter with a cutoff frequency of 10 Hz. Inverse dynamics analysis was performed using the Plug-in Gait full-body model, which is based on the Newton–Euler inverse dynamics approach, calculating joint moments from segmental linear and angular motion data. Joint centers were automatically estimated according to the standard procedures of the Plug-in Gait model. The hip joint centers were determined based on a pelvic coordinate system defined by the relative positions of the bilateral ASIS (anterior superior iliac spine) and PSIS (posterior superior iliac spine) markers. The knee and ankle joint centers were estimated based on anatomical landmarks of the femur and tibia.

Reflective markers were placed on the following anatomical landmarks: ASIS, PSIS, mid-lateral thigh, lateral femoral epicondyle, mid-shank, lateral malleolus, head of the second metatarsal, and calcaneus.

Joint power was calculated as the product of joint moment and angular velocity. Positive mechanical work was then obtained by integrating joint power over time, including only those intervals in which both moment and angular velocity had the same sign.

### 2.3. Exercise Intervention

**Figure 3:**
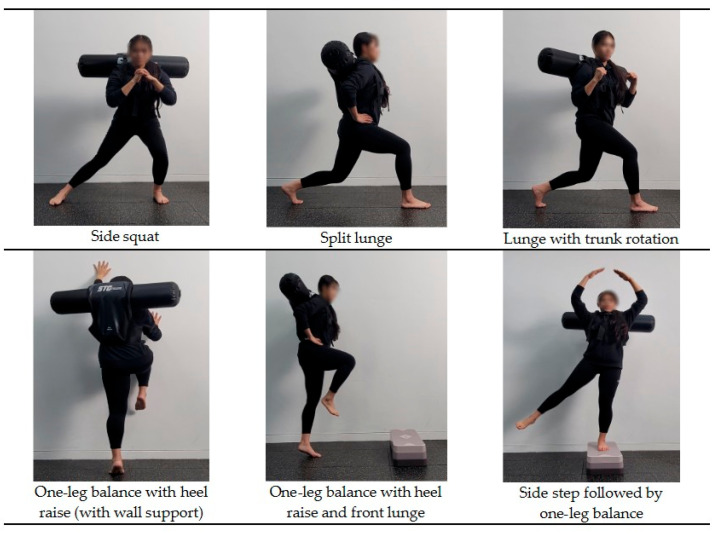
Representative exercises included in the dynamic stability training program, progressively applied over the 12-week intervention.

**Figure 4:**
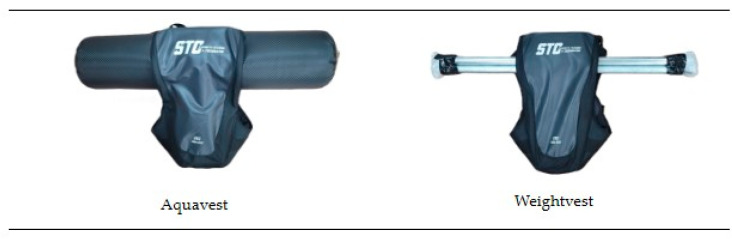
Structural differences between the aquavest and weighted vest used during the intervention.

The dynamic stability training (DST) in this study was based on the Instability Neuromuscular Training program proposed by Kang and Park [18] and was conducted twice a week for 12 weeks, totaling 24 sessions. Participants completed an average of approximately 23 out of 24 sessions (95% adherence). Each session consisted of a warm-up, main exercises, and a cool-down phase. The structure and details of the training program are presented in Table 2 and Figure 3. During weeks 1–6, participants performed low-intensity exercises focused on the bilateral stance and weight shifting (RPE 9–11, 3 kg). From weeks 7 to 12, the program progressed to moderate-intensity exercises involving a single-leg stance and balance maintenance (RPE 12–13, 4 kg). The experimental group wore an aquavest, while the control group wore a weighted vest during the same training protocol. The two vests were matched for total load, as well as for size and the wearing method. The key difference was in mass behavior: the aquavest contained freely moving water that created dynamic and multidirectional load shifts, whereas the weighted vest used fixed metal rods that provided a stable, unidirectional load without internal displacement, as illustrated in Figure 4.

Exercise intensity and progression were adjusted based on the participants’ rating of perceived exertion (RPE) [32,33].

### 2.4. Statistical Analysis

All data collected in this study were analyzed using IBM SPSS Statistics for Windows, version 25.0 (IBM Corp., Armonk, NY, USA). Means and standard deviations were calculated for all variables. To verify the homogeneity of demographic characteristics between groups, an independent t-test was performed. Prior to analyzing the intervention effects, the Shapiro–Wilk test was used to assess the normality of the data.

A mixed-design two-way ANOVA (group × time), with group as the between-subjects factor and time as the within-subjects factor, was used to examine the interaction effects of the intervention. When significant interaction or main effects were found, Bonferroni post hoc tests were conducted to identify differences across time points.

Effect sizes were calculated using eta squared (η^2^) and were interpreted as follows, based on Cohen’s guidelines: 0.01 = small, 0.06 = medium, and 0.14 = large.

## 3. Results

The results related to lower-limb-joint-moment- and positive-mechanical-work-,as well as spatiotemporal gait parameters, are presented in Table 3 and Table 4.

### 3.1. Changes in Lower-Limb Joint Moments and Ankle Positive Mechanical Work

The mixed-design two-way ANOVA revealed a significant main effect of time (*p* < 0.001) and a significant time × group interaction (*p* = 0.017) for the peak ankle plantarflexion moment. Post hoc analysis indicated that the aquavest group improved significantly across all time points, whereas the control group showed no improvement after week 6.

The Greenhouse–Geisser correction was applied to the peak hip extension moment due to a violation of sphericity; however, no significant changes were observed (*p* > 0.05).

For ankle positive mechanical work, the Greenhouse–Geisser correction was applied due to the violation of sphericity. After applying the correction, both the main effect of time (*p* < 0.001) and the time × group interaction (*p* < 0.001) were found to be significant. The aquavest group demonstrated consistent improvements over time, while no significant changes were observed in the control group (all *p* > 0.05).

The post hoc power analysis for the ankle plantarflexion moment showed a large time main effect (f = 0.63, power = 0.999), indicating sufficient sensitivity. In contrast, the group × time interaction effect was small (f = 0.17, power = 0.405), suggesting that the nonsignificant result may be due to limited power. For positive mechanical work, the time main effect demonstrated a large effect size (f = 0.55, power = 0.998), while the group × time interaction showed a moderate effect size (f = 0.32, power = 0.816), slightly exceeding the conventional threshold for sufficient power.

### 3.2. Change in Gait Parameters

The mixed-design two-way ANOVA revealed significant main effects of time in all gait parameters—cadence, stride length, and walking speed (all *p* < 0.001). Significant time × group interactions were observed for stride length (*p* < 0.001) and gait speed (*p* < 0.001), but not for cadence (*p* = 0.818).

In the aquavest group, post hoc tests indicated consistent improvements in stride length and gait speed across all time points, while cadence increased significantly only from baseline to week 12 (*p* = 0.020). In contrast, the control group showed significant changes in all three parameters only from baseline to week 12, with no significant differences observed between weeks 6 and 12 (all *p* > 0.05).

The post hoc power analysis for spatiotemporal gait variables showed that the time main effect for cadence had a moderate effect size (f = 0.33) and high power (0.938). For stride length, the time main effect yielded a large effect size (f = 0.68, power = 1.000), and the group × time interaction effect showed a moderate effect size (f = 0.35) with high power (0.961). For walking speed, the time main effect also demonstrated a large effect size (f = 0.73) and maximal power (1.000), while the group × time interaction showed a moderate effect size (f = 0.32) and sufficient power (0.923). These results indicate that all three variables had sufficient statistical sensitivity to detect both time-dependent changes and group-related interaction effects.

## 4. Discussion

This study aimed to investigate the effects of training with water inertia load on gait control by focusing on changes in lower-limb joint moments during gait in older women. Age-related decline in neural and musculoskeletal function is known to reduce gait automaticity and increase the cognitive and physical demands required to maintain balance, often resulting in a slower walking speed and higher energy expenditure [3,4,6,8]. These changes highlight the importance of interventions that can support more efficient and adaptive gait strategies in older adults. The aquavest induces unpredictable, multidirectional loading through the free movement of water within the device during motion. This dynamic and internally destabilizing stimulus requires greater postural and balance adjustments than traditional weight-based resistance [17,18,19]. Although neuromuscular mechanisms were not directly measured in this study, such conditions may have contributed to increased sensorimotor engagement and the refinement of balance strategies. Participants in the aquavest group demonstrated significantly greater improvements in gait-control variables compared to those in the weighted vest group, suggesting that training under dynamic perturbation may be beneficial for enhancing functional gait performance in older women.

Changes in lower-limb joint moments served as a key indicator in explaining modifications in gait strategy. In the aquavest group, the ankle plantarflexion moment significantly increased over time, indicating an enhanced push-off capability. Although the hip extension moment did not show significant changes, a slight decreasing trend may suggest a shift away from the compensatory proximal strategies typically observed in older adults [34,35]. These findings align with previous evidence that emphasizes the importance of restoring distal joint function to improve gait efficiency. Mackey and Robinovitch [36] reported that, in older women, the neural response speed is more critical than ankle strength for balance recovery, which is associated with reduced signal transmission at the spinal level and decreased motor unit recruitment [37]. Additionally, Franz [34] found that a faster contraction velocity of the plantar flexors leads to greater moment generation at foot-off, supporting the current findings. Moreover, the training program in this study included exercises such as side steps, side squats, and split lunges, which likely facilitated coordination between the ankle and hip musculature and improved the speed and stability of weight shifting [38,39]. Older adults often exhibit delayed weight transfer due to weakened hip abductor and adductor function [39], and this intervention may have helped compensate for such limitations. Lanza et al. [40] also reported that the faster neuromuscular activation of hip muscles is associated with shorter weight-transfer times, suggesting that the current training may have been effective in enhancing reflexive responses and inter-joint coordination. In contrast, the control group trained under a predictable and fixed-load environment, which likely limited sensorimotor feedback and neuromuscular activation. This aligns with previous studies showing that adaptation responses are reduced in predictable training conditions [41,42]. Indeed, the control group showed significant improvements only between 0 and 6 weeks, and 0 and 12 weeks, with no further gains observed from 6 to 12 weeks, indicating that unpredictable load characteristics may be critical for sustained neuromuscular adaptation.

An increase in the positive work generated at the ankle joint is interpreted as a key indicator of enhanced propulsion and improved energy efficiency during gait. In the aquavest group, positive work significantly increased across all time points, reflecting a strengthening of the ankle strategy and a reduced reliance on compensatory hip involvement. During gait, as body weight transfers from one limb to the other, the leading leg absorbs the impact while the trailing leg generates propulsion to counterbalance it. When propulsion at foot-off is insufficient, energy loss increases, ultimately reducing gait efficiency [35,43]. From this perspective, the increase in positive ankle work observed in the aquavest group suggests improvements in weight transfer and propulsion strategies, which subsequently led to enhanced gait speed, step length, and rhythm stability. Indeed, the aquavest group demonstrated significant improvements in cadence, gait velocity, and step length, with an average gait speed increase of 0.19 m/s. This increase exceeds the threshold for clinically meaningful change of 0.10 m/s proposed by Hortobágyi and Perera [44,45]. Previous studies have emphasized that even modest increases in gait speed are strong indicators of improved functional mobility and overall health in older adults. Notably, Hortobágyi et al. suggested that an improvement of ≥0.10 m/s may compensate for approximately 48–66% of age-related gait decline, highlighting its value as a clinical indicator of functional preservation [44]. Therefore, the 0.19 m/s increase observed in the aquavest group represents not only a statistically significant improvement, but also a clinically meaningful enhancement in mobility, daily functional capacity, and potentially, healthspan. In contrast, the control group showed a smaller increase of 0.08 m/s, indicating a more limited level of neuromuscular adaptation.

Gait speed is determined by a combination of cadence and stride length, both of which are closely related to neuromuscular control, balance maintenance, and propulsion generation [27,28]. Older adults, due to their reduced muscle strength and difficulty maintaining balance, tend to prefer increasing cadence over stride length as a strategy to improve gait speed [46]. In the present study, weight-shifting tasks such as front lunges and side walking were included as part of the training. These movements are similar to the muscle activation strategies used during the late stance phase of gait [47], requiring both forward propulsion and stability during landing. Such exercises likely enhanced the ability to shift the center of mass forward, contributing to increased stride length. In particular, performing these weight-shifting tasks under irregular perturbation conditions likely demanded a higher level of balance control and neuromuscular response in the aquavest group. The progressive increase in task difficulty and load after week 6 of training further stimulated improvements in response speed. These adaptations are thought to have played a key role in improving stride length during the latter part of the gait cycle [3,47,48,49]. Additionally, the non-linear loading stimuli provided by the training induced the repeated contraction and relaxation of the ankle muscles, which enhanced reaction speed and inter-joint coordination. This improvement in the weight-transfer strategy—from single-leg support to contralateral propulsion—likely contributed to the observed increases in gait speed and stride length. Kim et al. [50] also reported that older women with higher gait speeds tend to exhibit better muscular function and greater gait consistency, which aligns with the observed improvements in gait speed and rhythm control in the aquavest group in this study.

Therefore, training with water inertia load may effectively contribute to the restoration of ankle-based strategies and serve as a valuable intervention for improving gait function in older adults. However, the reduction in hip moment was not substantial, which may reflect the inherent difficulty older adults have in modifying established gait patterns. Fukuchi et al. [51] reported that older individuals tend to maintain their habitual motor strategies even when gait speed increases. This phenomenon is likely due to the slower rate of neuromuscular adaptation and the long-term habituation of movement patterns. Accordingly, to facilitate more efficient coordination between the ankle and hip joints, long-term approaches or training strategies specifically targeting inter-joint coordination may need to be incorporated.

This study was conducted based on two primary hypotheses, and the results largely supported both. First, the aquavest group showed significant improvements in most spatiotemporal gait variables, including gait speed, stride length, and cadence, indicating that the training effectively enhanced gait rhythm and propulsion. Second, the ankle plantarflexion moment and positive mechanical work significantly increased over time, while the compensatory hip moment tended to decrease. Although the reduction in hip moment was modest, this trend may still reflect a shift in the joint loading distribution toward a more efficient pattern across the lower limbs. These findings generally support the two hypotheses proposed in this study and demonstrate that training with water inertia load can have a positive impact on gait strategies and joint biomechanics in older adults.

This study has the following limitations:

1. The training intensity was regulated solely based on subjective RPE, without the use of objective physiological indicators.
2. The relatively small sample size may limit the generalizability of the findings to broader populations.

Although the post hoc power analysis revealed that most variables—including spatiotemporal gait parameters and positive mechanical work—achieved sufficient statistical power to detect both main effects and interaction effects, the ankle plantarflexion moment exhibited limited power for the group × time interaction (f = 0.17, power = 0.405), despite a strong main effect. This suggests that some nonsignificant interaction results may be attributed to insufficient power rather than the absence of actual effects. Additionally, participant dropout during the intervention period reduced the final sample size, which may have further limited statistical sensitivity. These factors underscore the importance of conducting future studies with larger and more stable samples to ensure robust statistical power and broader applicability of the findings.

## 5. Conclusions

This study investigated the effects of dynamic stability training using water inertia load on gait strategies and lower-limb joint biomechanics in older women. The results demonstrated improvements in spatiotemporal gait variables, functional recovery of the ankle joint, and a more efficient distribution of joint moments across the lower limbs.

These findings suggest that restoring the ankle strategy has a meaningful impact on gait efficiency and propulsion, highlighting the clinical applicability of this intervention. Furthermore, the two hypotheses proposed in this study—(1) improvements in gait speed, stride length, and cadence, and (2) an increased ankle plantarflexion moment and positive mechanical work with a reduction in the compensatory hip moment—were generally supported. This indicates that training with water inertia load can contribute to enhancing gait strategies in older adults. However, the reduction in the hip joint moment was limited, highlighting the need for future studies to develop long-term and structured intervention programs aimed at improving inter-joint coordination.

## Figures and Tables

**Table 1:** Characteristics of study participants (n = 24).

	AG (n = 12)	CG (n = 12)
Age (years)	67.83 ± 2.41	68.67 ± 3.52
Weight (kg)	59.63 ± 11.09	60.70 ± 10.37
Height (cm)	157.17 ± 5.01	156.08 ± 4.22
BMI (kg/m^2^)	24.35 ± 1.05	24.61 ± 1.30

Values are presented as means ± standard deviations. AG, aquavest group; CG, control group.

**Table 2:** Dynamic stability training program.

Training	0~6 Weeks	7~12 Weeks	Time
Intensity	9~11 RPE	12~13 RPE	
	with weight (3 kg)	with weight (4 kg)	
	2 sets	1 set	
Warm-up	Hip and ankle joint mobility and spine stretch	Hip and ankle joint mobility and spine stretch	10 min
DST exercise	Squat	Squat and heel raise Balance	30 min
	Squat and heel raise	Side squat followed by one-leg balance	
	Side squat	Front lunge (dynamic speed)	
	Split lunge	Back lunge (dynamic speed)	
	Lunge with trunk rotation	Front lunge with trunk rotation (dynamic speed)	
	Lunge (with step box)	One-leg balance with heel raise (with wall support)	
	Side squat (with step box)	One-leg balance with heel raise and front lunge (with step box, dynamic speed)	
	Walking (with step box)	Side step followed by one-leg balance (with step box, dynamic speed)	
	Side walking (with step box)	Side step jump over the step box (dynamic speed)	
Cool down	Cat stretch	Cat stretch	10 min
	Spine stretch	Spine stretch	
	Bear position	Bear position	

**Table 3:** Changes in ankle and hip joint moments and ankle positive mechanical work.

Variable	Group	0 Weeks	6 Weeks	12 Weeks	Source	*p*	η^2^	Post Hoc
Ankle plantarflexion (Nm/kg)	AG	−0.43 ± 0.36	−0.61 ± 0.26	−0.95 ± 0.30	Time	<0.001	0.63	0–6 weeks: 0.034
								0–12 weeks: <0.001
								6–12 weeks: <0.001
	CG	−0.38 ± 0.18	−0.64 ± 0.35	−0.70 ± 0.24	Group × Time	0.017	0.17	0–6 weeks: <0.01
								0–12 weeks: <0.001
								6–12 weeks: 1.000
Hip extension (Nm/kg)	AG	−7.44 ± 3.40	−6.21 ± 2.80	−6.05 ± 2.61	Time	0.481		ns
	CG	−5.60 ± 3.60	−5.70 ± 2.80	−5.82 ± 2.82	Group × Time	0.329		ns
Ankle positive work (J/kg)	AG	2.14 ± 1.74	3.35 ± 1.91	5.63 ± 2.12	Time	<0.001	0.55	0–6 weeks: <0.01
								0–12 weeks: <0.001
								6–12 weeks: <0.01
	CG	1.46 ± 0.96	2.18 ± 0.84	2.37 ± 0.92	Group × Time	<0.001	0.32	0–6 weeks: 0.066
								0–12 weeks: 0.087
								6–12 weeks: 1.000

Note: Data are presented as mean ± standard deviation. AG: aquavest group; CG: control group. ns: not significant. *p*-values are based on mixed-design two-way ANOVA. Post hoc comparisons were adjusted using the Bonferroni correction. η^2^: partial eta squared (small = 0.01, medium = 0.06, large ≥ 0.14).

**Table 4:** Changes in gait parameters.

Variable	Group	0 Weeks	6 Weeks	12 Weeks	Source	*p*	η^2^	Post Hoc
Cadence (step/min)	AG	118.80 ± 8.85	121.86 ± 8.08	126.28 ± 7.00	Time	<0.001	0.33	0–6 weeks: 0.751
								0–12 weeks: 0.020
								6–12 weeks: 0.106
	CG	118.73 ± 10.32	121.49 ± 9.59	124.51 ± 7.67	Group × Time	0.818	0.01	0–6 weeks: 0.478
								0–12 weeks: 0.044
								6–12 weeks: 0.274
Stride length (m)	AG	0.75 ± 0.05	0.81 ± 0.05	0.88 ± 0.05	Time	<0.001	0.68	0–6 weeks: 0.011
								0–12 weeks: <0.001
								6–12 weeks: <0.01
	CG	0.75 ± 0.03	0.79 ± 0.05	0.80 ± 0.04	Group × Time	<0.001	0.35	0–6 weeks: <0.01
								0–12 weeks: 0.011
								6–12 weeks: 1.000
Walking speed (m/s)	AG	0.74 ± 0.07	0.83 ± 0.06	0.93 ± 0.06	Time	<0.001	0.73	0–6 weeks: <0.01
								0–12 weeks: <0.001
								6–12 weeks: <0.001
	CG	0.74 ± 0.07	0.80 ± 0.09	0.82 ± 0.08	Group × Time	<0.001	0.32	0–6 weeks: <0.01
								0–12 weeks: <0.01
								6–12 weeks: 0.866

Note: Data are presented as mean ± standard deviation. AG: aquavest group; CG: control group. *p*-values are based on mixed-design two-way ANOVA. Post hoc comparisons were adjusted using the Bonferroni correction. η^2^: Partial eta squared (small = 0.01, medium = 0.06, large ≥ 0.14).

## Data Availability

The data used in this study are available upon reasonable request and will be deposited in a public repository upon publication.
